# Quantitative Hemodynamic Measurements in Cortical Vessels Using Functional Ultrasound Imaging

**DOI:** 10.3389/fnins.2022.831650

**Published:** 2022-04-12

**Authors:** Clément Brunner, Emilie Macé, Gabriel Montaldo, Alan Urban

**Affiliations:** ^1^Neuro-Electronics Research Flanders, Leuven, Belgium; ^2^VIB, Leuven, Belgium; ^3^Imec, Leuven, Belgium; ^4^Department of Neuroscience, Faculty of Medicine, KU Leuven, Leuven, Belgium; ^5^Brain-Wide Circuits for Behavior Research Group, Max Planck Institute of Neurobiology, Martinsried, Germany

**Keywords:** functional ultrasound imaging, cerebral blood flow (CBF), cerebral blood volume (CBV), neurovascular coupling (NVC), red blood cell velocity (RBCv)

## Abstract

Red blood cell velocity (RBCv), cerebral blood flow (CBF), and volume (CBV) are three key parameters when describing brain hemodynamics. Functional ultrasound imaging is a Doppler-based method allowing for real-time measurement of relative CBV at high spatiotemporal resolution (100 × 110 × 300 μm^3^, up to 10 Hz) and large scale. Nevertheless, the measure of RBCv and CBF in small cortical vessels with functional ultrasound imaging remains challenging because of their orientation and size, which impairs the ability to perform precise measurements. We designed a directional flow filter to overpass these limitations allowing us to measure RBCv in single vessels using a standard functional ultrasound imaging system without contrast agents (e.g., microbubbles). This method allows to quickly extract the number of vessels in the cortex that was estimated to be approximately 650/cm^3^ in adult rats, with a 55–45% ratio for penetrating arterioles versus ascending venules. Then, we analyzed the changes in RBCv in these vessels during forepaw stimulation. We observed that ∼40 vessels located in the primary somatosensory forelimb cortex display a significant increase of the RBCv (median ΔRBCv ∼15%, maximal ΔRBCv ∼60%). As expected, we show that RBCv was higher for penetrating arterioles located in the center than in the periphery of the activated area. The proposed approach extends the capabilities of functional ultrasound imaging, which may contribute to a better understanding of the neurovascular coupling at the brain-wide scale.

## Introduction

The cerebral vasculature is an active system that continuously regulates the supply of blood to maintain the physiological state of the brain while adequately supporting cerebral functions (Roy and Sherrington, 1890; Iadecola, 2017). Our understanding how the brain blood supply is regulated, affected, or adapts in pathologies (e.g., stroke, Alzheimer’s Disease, or small vessel diseases) remains incomplete. Thus, providing an accurate measurement of hemodynamic features such as cerebral blood volume (CBV), cerebral blood flow (CBF), and red blood cell velocity (RBCv) in cerebral vessels at the sub-second and brain-wide scale would help deepen our knowledge of cerebrovascular changes in health and disease.

Optical neuroimaging methods including laser speckle contrast imaging, two-photon microscopy, and optical coherence tomography have been preferred in investigating brain hemodynamics as they enable measurements of RBCv (Kleinfeld et al., 1998; Chaigneau et al., 2003, 2019; Shih et al., 2012) and/or CBF (Hillman, 2007; Devor et al., 2012; Li et al., 2020) in the brain vessels. However, optical neuroimaging strategies are mostly restricted to the brain surface and cortical vessels with a coverage of a few individual vessels.

In clinic, ultrasound Doppler is the most used method to measure the blood velocity deep in the body. However, conventional ultrasound scanners could only address large vessels of a few millimeters in diameter (Rohren et al., 2003; Lee, 2014) while excluding the small vessels for which the flow cannot be detected. Such a limitation has been overcome over the last decade with the development of new ultrasound techniques, namely functional ultrasound imaging and ultrasound localization microscopy.

Functional ultrasound imaging offers a brain-wide imaging of the CBV dynamics at the subsecond scale with submillimetric resolution (Macé et al., 2011, 2013; Brunner et al., 2021). The ultrasound principle employed here relies on an improved Doppler method that increases the sensitivity to detect signal small vessels. Indeed, the brain is illuminated with ultrasound plane-waves at high frame rate (∼500 Hz), a compound method that increases the signal-to-noise ratio when compared with standard approaches (Montaldo et al., 2009). However, the quantification of the RBCv remains difficult with such approach. First, the Doppler effect relies on the angle between the vessel and the ultrasound beam that is not easy to measure for small vessels. Second, the measure of the Doppler frequency is very sensitive to noise and is unstable in voxels with low signal or composed with vessels of opposite directional flow. Third, the blood velocity is uniformly distributed along the vessel with the maximum velocity at the center and lower speed near the vessel wall. This adds extra difficulty when analyzing the broad spectrum of the Doppler signal from a single voxel containing several velocities from the vessel.

Ultrasound localization microscopy enables the precise measurement of the blood velocity in small vessels (Errico et al., 2015; Lowerison et al., 2022). This method relies on the individual tracking of gas-filled microbubbles providing a measurement of the blood cell velocity in surface and cortical vessels down to the second order of penetrating artery branching (Errico et al., 2015). However, the short half-life of injected microbubbles (Paefgen et al., 2015) strongly limits the capacity to robustly quantify repeated hemodynamic changes over time. Using a contrast-free strategy based on speckle decorrelation, Tang and colleagues (Tang et al., 2020) recently reported on quantitative ultrasound cerebral blood flow velocimetry of mouse brain functions with higher accuracy than existing power-Doppler and color-Doppler ultrasound strategies.

In this study, we exploit functional ultrasound imaging acquisition data to extract a reliable RBCv information without the need of contrast agent or substantial modifications in the functional ultrasound imaging acquisition procedure. Using an angiographic scan of rat brain, we segmented the cortical vessels with enough signal to correctly quantify the blood flow direction and Doppler spectrum to extract the average RBCv in individual vessels using a simplified model of laminar flow. Then, we report on the absolute RBCv and relative CBF changes of individual cortical vessels under resting-state and evoked conditions. By providing quantitative hemodynamic measurements, the functional ultrasound imaging modality extends the toolset for studying the neurovascular coupling at the brain-wide scale and provides new insights into neurological diseases.

## Materials and Methods

### Animals

Experimental procedures were approved by the Committee on Animal Care of the Catholic University of Leuven, in accordance with the national guidelines on the use of laboratory animals and the European Union Directive for animal experiments (2010/63/EU). Adult male Sprague–Dawley rats (*n* = 3; Janvier Labs, France) weighing 200–300 g were used for this study and were kept in a 12-h dark/light cycle environment at a temperature of 22°C with *ad libitum* access to food and water.

### Cranial Window for Brain-Wide Imaging

A cranial window was performed under isoflurane anesthesia (Iso-Vet, Dechra, Belgium) continuously delivered at 0.6 L/min through a nose mask. A mixture of 5% isoflurane in compressed dry air was used for induction of anesthesia, subsequently reduced to 2.0–2.5% during surgery and to 1.5% for imaging. Body temperature was monitored using a rectal probe and maintained at 36.5 ± 0.5°C using a digitally controlled heating blanket (CODA; Kent Scientific Corp., United States). Intraperitoneal injection of 5% glucose solution was provided every 2 h to prevent dehydration. Xylocaine (0.5%; AstraZeneca, England) was injected subcutaneously into the head skin as pre-operative analgesia. The scalp was shaved and cleaned with iso-betadine before being removed over the entire dorsal skull. The cranial window extended from bregma +3.5 to −5.5 mm and laterally ± 6.0 mm from midline (Brunner et al., 2020). Sterile saline was regularly added during drilling sessions to avoid overheating of the tissue. The skull was carefully removed without damaging the dura adapting the surgical procedure described by Brunner et al. (2021) and covered with a layer of 2% agarose (Sigma-Aldrich, United States).

### Forepaw Electrical Stimulation

Two needle electrodes were inserted into the palmar skin of the left forepaw to generate an electrical stimulus (200 μs pulse width, 1.5 mA intensity, 5 Hz repetition frequency) through a constant current isolator (DS3; Digitimer Ltd, United Kingdom). Each trial consisted in a pre-stimulus/baseline period of 10 s followed by a 5-s stimulation period (25 electric pulses at 5 Hz) and a 9-s post-stimulus period for a total duration of 24 s, with a minimal inter-trial period of 1 min.

### Functional Ultrasound Imaging Set-Up

**FIGURE 1:**
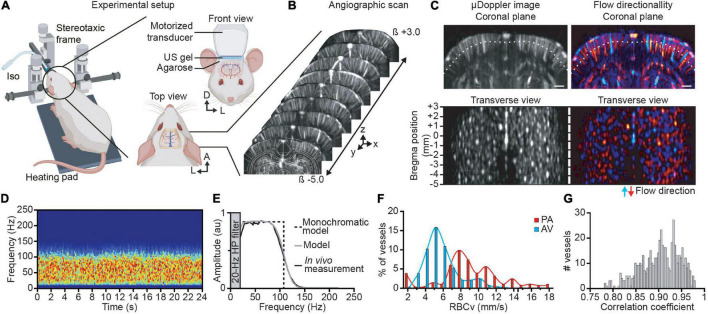
Brain-wide angiographic scan and single vessel velocimetry. **(A)** Experimental set-up for brain-wide functional ultrasound imaging in isoflurane-anesthetized rats. **(B)** Antero-posterior cerebral μDoppler images scan (i.e., angiographic scan) from bregma (β) +3 to −5 mm overlaid with corresponding brain atlas plane (white outlines). X, y, and z are the axis convention of the μDoppler image. **(C)** Original and color-coded coronal μDoppler image (top). The red range of intensities depicts the positive part of the Doppler spectrum corresponding to the blood flowing down in penetrating arteries, while the negative part of the Doppler spectrum, i.e., blood flowing up in ascending venules, is colored in the blue range. The white dotted line represents the section used for the transverse view representation located 0.5-mm deep down the cortical surface. Each spot corresponds to a sectioned cortical vessel before (left) and after directionality discrimination (right). **(D)** Spectrogram of a single cortical vessel showing a broadband and uniform signal in a control trial (no forepaw stimulation delivered). **(E)** Average spectrum of the vessel (black curve) showing a flat spectrum band from 25 to 80 Hz, monochromatic model (black dotted line) representing a simple square function, and the broadband model (gray curve) considering the 20-Hz high-pass filter and the transducer bandwidth model correctly fits the experimental data. **(F)** Distribution of the red blood cell velocity (RBCv) in cortical penetrating arterioles (PA; in red) and ascending venules (AV; in blue). **(G)** Correlation coefficient between the broadband model spectrum and the experimental data of all cortical vessels. A, anterior; D, dorsal; L, left. Scale bar = 1 mm. Created with BioRender.com.

We used an ultrasound linear transducer (15 MHz, 128 elements, 60% bandwidth, 0.1 mm pitch, L15–128; Vermon, France) linked to a 128-channel emission-reception electronics (Vantage, Verasonics, United States) and controlled by a high-performance computing workstation equipped with 4 GPUs (AUTC, fUSI-2, Estonia). The transducer can image a single plane section of 12.8-mm width and 10-mm depth and is oriented to image in the coronal section. To scan the full brain, the ultrasound probe is mounted on a motorized linear stage (T-LSM200A; Zaber Technologies Inc., Canada). The experimental set-up and axis convention are shown in Figures 1A,B with the *x*-axis defined as the mediolateral orientation from the midline, the *y*-axis as the antero-posterior orientation originating at bregma point, and the *z*-axis as the dorso-ventral orientation. The probe is oriented in the _*x*_ direction to acquire images in the _(x,z)_ plane. The motorized linear stage scans the brain in the _*y*_ direction. The acoustic coupling between the brain and the probe is ensured by a layer of ultrasound gel (Aquasonic Clear; Parker Laboratories Inc., United States). Imaging is carried out on an anti-vibration table to minimize external sources of vibration. Software for the data acquisition is open access and can be found in Brunner et al. (2021).

### Angiographic and Functional Scans

Prior to functional stimulation, an angiographic scan of the brain consisted in consecutive μDoppler images acquired every 100 μm from bregma +3.0 to −5.0 mm (i.e., 80 μDoppler images). Each plane was recorded in 0.4 s at 750 Hz generating a set of 300 compound images computed in one μDoppler image; see Brunner et al. (2021) for details. To reduce the noise, each plane was averaged 10 times. To record hemodynamic changes in response to forelimb stimulation, 18 planes spaced by 300 μm covering the entire somatosensory forelimb cortex were imaged (from bregma +2.1 to −3.0 mm). The functional stimulation was repeated three times per plane. Each trial lasted 24 s with a frame rate of 750 Hz generating a set of 18,000 images. More details about the ultrasound sequences used here are included in Table 1. In both angiography and functional scans, the output is a 4-dimensional data _*a(x,  y,  z,  t)*_where _*x,y*_, and_  z _ are the spatial dimensions and _*t*_ is the time sampled at 750 Hz.

**TABLE 1:** Parameters of angiographic and functional scans.

	Parameters	Angiographic scan 3D angiography	Functional scan Velocity measurement
μDoppler image	Number of angles	13	5
	Angles/step	−12° to 12°/2°	−12° to 12°/6°
	Repetition per step	3	3
	Firing frequency	9,750 Hz	11,250 Hz
	Frame rate	750 Hz	750 Hz
	Number of images	300	18,000
	Acquisition time	0.4 s	24 s
Scan	Volume scanned	Bregma +3.0 to −5.0 mm	Bregma +2.1 to −3.0 mm
	Imaging step	150 μm	300 μm
	Number of planes	80	18

### Processing

Each image _*a(x,  y,  z,  t)*_ is composed of a signal coming from the blood *b* and tissue *Ti* such as *a*(*x,z,t*) = *b*(*x,z,t*)*+Ti*(*x,z,t*). To obtain the blood component *b*(*x,z,t*) (also called Doppler signal), a 20-Hz high-pass filter was applied on all acquired images to remove the tissue part (slower movements) from the blood signal. The 3D angiographic images and individual vessel spectrums were computed using the blood component *b*(*x,z,t*).

### Blood Filtering

The raw images obtained from the previous step are composed from a combination of the blood and the tissue signals *a* = *b* + *Ti*, where _*b*_ is blood and *Ti* is the tissue signal. The latter is eliminated by applying a 20-Hz high-pass filter that removes the lower frequency from tissue, while conserving the blood signal also called the Doppler signal:


(1) b⁢(x,y,z,t)=F⁢i⁢l⁢t⁢e⁢r⁢H⁢P⁢(a⁢(x,y,z,t))

### Angiographic Images

We perform an angiographic image to identify the position and orientation of the vessels. The angiographic image was computed as the total intensity in each pixel and estimator of the relative rCBV (Macé et al., 2011, 2013; Brunner et al., 2021):


(2) $$I\,\left(x,y,z\right)=\int {\left|b\,\left(x,y,z,t\right)\right|}^{2}\,d\,t$$

To determine the direction of the flow in each vessel (blood going toward the probe or away from the probe), we compute a directional angiographic image by splitting the intensity in positive and negative frequencies as previously described (Macé et al., 2013):


(3) $$\begin{array}{c} I^{+}\left(x,y,z\right)=\int_{0}^{+\infty }|B\left(x,y,z,\omega {|}^{2}d\omega \right)\\ I^{-}\left(x,y,z\right)=\int_{-\infty }^{0}|B\left(x,y,z,\omega {|}^{2}d\omega \right) \end{array}$$

where _*B(x,y,z,ω)*_ is the Fourier transform of _*b(x,y,z,t)*_.

### Single-Vessel Identification

Individual cortical vessels were identified using two-step procedures. First, we converted the coronal stack into a 3D stack allowing us to visualize it in the coronal, sagittal, and transversal directions (Brunner et al., 2022). Then, we extracted each vessel while being positioned in a transversal view in the middle of the cortex (Figure 1C). Finally, we used the coronal view to select two points in a region where the vessel has a straight shape. These points were also used to calculate the vessel’s angle related to the ultrasound beam.

### Single-Vessel Intensity

The intensity of an individual vessel was then computed by averaging the intensity of the Doppler signal in all the voxels from the same vessel:


(4) $$I_{v}\,\left(t\right)=\int_{v\,e\,s\,s\,e\,l}{\left|b\,\left(x,t,z,t\right)\right|}^{2}\,d\,v$$

where _*vessel*_ represents all the points _(x,y,z)_ of the same individual vessel. This intensity can be interpreted as a magnitude proportional to the CBV in this vessel.

### Single-Vessel Spectrogram

To extract the Doppler frequency at each time point, the Doppler signal has been computed by the sliding spectrogram:


(5) $$B\,t\,\left(x,y,z,\omega ,t\right)=\int b\,\left(x,y,z,t^{\prime }\right)\,W\,\left(\frac{t-t^{\prime }}{2\,T}\right)\,e^{i\,\omega \,t^{\prime }}\,dt^{\prime }$$

in which *W* is the square window function of length *T* = 180 ms.

In voxels without vessels or vessels too small to be detected (e.g., capillaries), the spectrogram remains inaccessible. To avoid such an issue, we computed an average spectrogram of all the voxels from the same vessel that can be identified within the angiographic image. The spectrogram from a single vessel is defined as


(6) $$s\,t_{v}\,\left(\omega ,t\right)=\int_{v\,e\,s\,s\,e\,l}{\left|B\,t\,\left(x,y,z,\omega ,t\right)\right|}^{2}\,d\,v$$

Note that the spectrogram for each voxel in the vessel is computed and its absolute value is measured before averaging to avoid random phase cancellation between different voxels.

Under resting-state condition, we can define the average spectrum in the vessel as


(7) $$s_{v}\,\left(\omega \right)=\int s\,t_{v}\,\left(\omega ,t\right)\,d\,t$$

### Activity Map

The activity map was computed as the correlation of the stimulus with the blood signal, as previously described (Macé et al., 2011; Urban et al., 2014, 2015; Brunner et al., 2018, 2021):


(8) $$c\,\left(x,y,z\right)=\frac{\int \left|b\,\left(x,y,z,t\right)\right|\,s\,t\,i\,m\,\left(t\right)\,d\,t}{\sqrt{\int {\left|b\,\left(x,y,z,t\right)\right|}^{2}\,d\,t\,\int s\,t\,i\,m^{2}\,\left(t\right)\,d\,t}}$$

with *stim*(*t*) as the period of stimulation. A vessel is considered “active” when the RBCv increases by more than 2 times the baseline’s standard deviation during the stimulation period.

### *In vitro* Model

We simulate vessels with tubes of polyamide imbibed in a block of 2% agarose. We tested three different tubing with an inner diameter of 104, 178, and 254 μm (Microlumen, United States). A blood-mimicking fluid (CIRS, United States) was flowed continuously with a pump with different fluid velocities ranging from 4 to 35 mm/s. The angle of one tube (254 μm diameter) and the ultrasound beam was changed from 12 to 50° keeping a constant fluid velocity.

## Results

In this study, relative cerebral blood volume (rCBV), relative cerebral blood flow (rCBF), and red blood cell velocity (RBCv) were quantified in individual cortical vessels under resting-state and evoked conditions. Cortical vessels were identified and classified as penetrating arterioles or ascending venules depending on their orientation away or toward the transducer, as shown on the Doppler spectrum. A model to quantify the RBCv was then established *in vitro* and confirmed *in vivo* by experimental data. Finally, the rCBV, rCBF, and RBCv dynamics were addressed in individual vessels by means of a functional stimulation.

### Brain-Wide Imaging of the Cortical Vasculature

Brain-wide ultrasound imaging was carried out in head-fixed anesthetized rats directly after being subjected to cranial window surgery (Figure 1A). An angiographic scan composed of 80 μDoppler coronal images spaced by 150 μm was performed covering the imaging window (Figure 1B). From the volumetric angiographic scan, a transverse view has been computed 0.5-mm deep down the cortex surface (Figure 1C; white dotted line) enabling the identification of 515 individual vessels within 80-mm^2^ surface deep in the cortex (Figure 1C, left). The angiographic image has been split in two directional components *I*^+^ and *I*^–^ [see Eq. (3)] allowing for the discrimination between vessels with a blood flow going up toward the ultrasound probe (*I*^+^) or flowing away from the brain surface (*I*^–^), respectively, assimilated to ascending venules (AV) and penetrating arterioles (PA) when considering the structural and functional organization of the cortical vasculature (Blinder et al., 2013; Kirst et al., 2020). Color-coded coronal μDoppler image and the computed transverse view show that 55% of the cortical vessels are PA and 45% AV (respectively, red and blue vessels, Figure 1C, right).

### Quantitative Measurement of Red Blood Cell Velocity in Single Vessels Using Doppler Spectra

To measure the Doppler frequency in single cortical vessels, we performed a recording of 24 s under resting-state conditions. The spectrogram depicting the temporal evolution of the Doppler spectrum extracted from an individual penetrating arteriole is stable along the recording period (Figure 1D). The temporal averaging of the Doppler spectrum of this vessel showed broadband ranging from 20 to 150 Hz with a plateau between 20 and 80 Hz (Figure 1E, black curve). We filtered frequencies below 20 Hz using a high-pass filter to remove the tissue part (see Materials and Methods). Note that the spectrogram from the penetrating arteriole differs from the one observed in larger vessels (e.g., carotid artery). Indeed, the latter shows a single-peak spectrogram at the corresponding Doppler frequency directly giving the blood velocity value (Rohren et al., 2003; Lee, 2014) whereas the spectrum from cortical vessels depicts a broadband spectrum with a “unique” Doppler frequency (Figure 1E). Thus, we cannot directly compute the blood velocity without using a complete model of the spectrum.

### Modeling the Spectrum

The broadband spectrum can be explained by the distribution of the blood velocities in small vessels. Indeed, the blood flow in cortical vessels can be considered as laminar with a distribution of velocity along the vessel cross-section relying on *v*(*r*) = *v*_0_(1−*r*^2^/*R*^2^) (i.e., Bernoulli equation), with *v*_0_ the maximal velocity in the center of the vessel and *R* the radius of the vessel, giving a mean velocity $\bar{v}=v_{0}/2$. As a single functional ultrasound imaging voxel (100 × 110 × 300 μm^3^) is larger than the lumen diameter of most of the cortical vessels (Shih et al., 2009; Driscoll et al., 2013), one single voxel integrates all the velocities from the imaged vessel. Moreover, by excluding the effects of the transducer bandwidth (i.e., supposing a monochromatic wave), we demonstrate that the spectrum is a square function with a maximal frequency ω_*max*_ = 2ω_*us*_*v*_0_*cos* θ/*c* (Supplementary Material).

Using a monochromatic model (Figure 1E, black dotted line), the mean RBCv can be computed in single vessels as


(9) v¯=ω¯⁢c/(2⁢ω⁢cosu⁢s⁡θ)

with $\bar{\omega }$ the mean frequency defined as $\bar{\omega }=\int s_{v}\,\left(\omega \right)\,\omega \,d\omega /\int s_{v}\,\left(\omega \right)\,d\omega$. Note that Eq. (9) is exactly the standard formula that links the velocity with the Doppler frequency.

Then, the model is improved by considering that (1) the higher frequency edge is smoothed by the transducer bandwidth and (2) the 0 Hz frequency edge is sharpened by a high-pass filter (see Supplementary Material and Censor et al., 1988 for details). By doing so, the broadband model correctly fits the experimental data with a correlation coefficient of 0.95 (Figure 1E, gray curve).

The smoothed high-frequency edge does not modify the mean frequency, while it induces a **_ω_cutt_** shift, necessary to correct as


(10) $$\bar{v}=\left(\bar{\omega }-\omega_{c\,u\,t\,t}\right)\,c/\left(2\,\omega_{u\,s}\,\cos\theta \right)$$

Note that Eq. (10) is only valid when the mean velocity within individual vessels is higher than ω_*cutt*_*c*/(2ω_*us*_*cos* θ); otherwise, the Doppler frequency is too low and removed by the high-pass filter.

**FIGURE 2:**
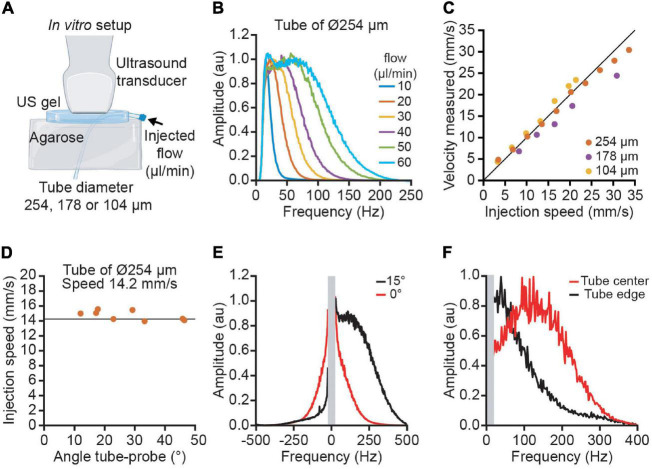
Quantitative velocity measurement performed *in vitro* using polyimide tubes of various diameters. **(A)** The experimental set-up used blood mimicking fluid injected in 3 tubes of increasing diameter (104, 178, and 254 μm) to simulate the variety of cortical vessels. **(B)** Average Doppler spectrum for different flows ranging from 10 to 60 μl/min. **(C)** Measurement of velocities using ultrasound imaging for the three tubes (yellow: 104 μm, purple: 178 μm, orange: 254 μm). **(D)** Example of the velocity measured at different angles ranging from 10 to 45° for the tube of 254 μm diameter. The vertical line corresponds to the actual speed of the particles (14.2 mm/s). **(E)** Effect of the spectral broadening. Due to the spectral broadening, a horizontal tube (angle 0 in red) has a positive and negative spectrum. With an angle of 15°, the Doppler effect is higher than the spectral broadening and the spectrum is lateralized. **(F)** Effect of the laminar flow profile. The spectrum in the center of the tube (red) has a higher frequency than in the edge (black).

This simplified model does not include the effect of spectral broadening. Indeed, when a flow of particles crossed the focal point perpendicularly to the ultrasound beam, the Doppler effect is 0. However, there is an enlargement of the spectrum due to variations in the amplitude of the signal (Figure 2E). In practice, this does not affect the mean frequency, but some part of the spectrum can span from negative to positive frequencies, an information lost after applying the directional filter. A vessel with an angle of >15° ensures that the spectrum is either entirely positive or negative and that the contribution of the Doppler shift on the Doppler spectrum is taking over the spectral broadening (see Supplementary Material).

### *In vitro* Validation

To confirm the validity of the model, we performed an *in vitro* calibration where we imposed different fluid speeds into artificial vessels while imaging with functional ultrasound (Figure 2A). The average spectrum from the artificial vessel shows high similarity with the *in vivo* experiment (Figure 2B). The spectrum has a square shape with a smoothed edge. The error between the measure velocity and the injected flow velocity is below 10% for the 3 tube diameters and a velocity range between 4 and 35 mm/s (Figure 2C). By changing the angle between the tube and the probe, we observed that the velocity measurement is independent from the angle for angles higher than 15° (Figure 2D). For low angles (< 15°), the spectral broadening results in a spectrum with both positive and negative frequencies; however, an angle of 15° has a Doppler shift high enough to place all the spectrum on one side (Figure 2E). To illustrate the effect of the velocity profile inside the tube, we compare the spectrum in the center of the tube with the edge. We observe that the central part has a higher frequency than the edge (Figure 2F). The integration of all the speeds in the tube explains the flat spectrum in the small vessels.

### Velocity Distribution in the Cortical Vessels

For all the 515 identified vessels in the cortex, we measured the RBCv using Eq. (10). The penetrating arterioles show higher velocities than the ascending venules (Figure 1F). It is important to note that the velocity measurement is only possible if the spectrum has a square shape (dotted line, Figure 1E). To verify this point, we have computed the correlation between the spectrum and its best theoretical fit for each vessel. We noticed that the correlation with the theoretical model ranged from 0.78 to 0.98, centered on 0.9 (Figure 1G). This correlation gives a quality factor to reject noisy vessels.

### Single Vessel Evoked Relative Cerebral Blood Volume, Red Blood Cell Velocity, and Relative Cerebral Blood Flow Variations

**FIGURE 3:**
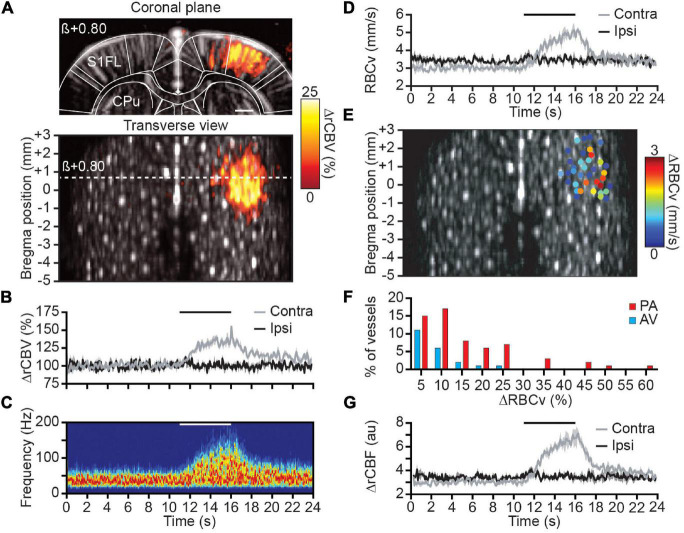
Spatial distribution and hemodynamic quantification of activated vessels. **(A)** Coronal μDoppler image (top panel) and transverse view (bottom panel) overlaid with the color-coded correlation map highlighting rCBV changes (%) in the contralateral S1FL region during forelimb stimulation. **(B)** Average rCBV increase in a single cortical S1FL vessel contralateral (gray) and ipsilateral (black) to the forepaw stimulation (horizontal black line). **(C)** Spectrogram (Hz) from a single penetrating arteriole located in the S1FL contralateral to the forepaw stimulation (horizontal white line). **(D)** RBCv (mm/s) changes in the same vessels than in **(B)**. **(E)** Spatial location of cortical vessels showing RBCv changes (%) in response to forelimb stimulation. The distribution of RBCv increase is ranging from less than 1 mm/s up to 3 mm/s. **(F)** Distribution of the RBCv increase for all activated vessels. **(G)** rCBF (au) changes in the same vessels than in **(B)**. All traces are mean ± sem of 3 trials. S1FL, primary somatosensory cortex of the forelimb; CPu, caudate putamen; β, bregma; scale bar = 1 mm.

To address the RBCv variation in individual cortical vessels, we studied the functional changes by the means of somatosensory stimulation of the forepaw (see Materials and Methods). First, we analyzed the spatial distribution of the activated region by computing the correlation map of rCBV change with the stimulation paradigm (see Eq. 7) for each coronal plane and performing the 0.5-mm depth transverse view (Figure 3A). Second, the stimulation-induced activity map (averaged of 3 trials/plane) was localized within the primary somatosensory cortex of the forelimb (S1FL), contralaterally to the stimuli, with a regional rCBV increase up to ∼25% of the baseline level. Moreover, the time course of rCBV variation from a single penetrating arteriole located within the contralateral S1FL also shows a ∼25% increase of signal whereas no variation has been detected in individual vessels located ipsilaterally to the stimulus (respectively, gray and black plots, Figure 3B).

Second, the spectrogram from the same contralateral vessel was extracted and depicts a frequency increase during the forepaw stimulation (Figure 3C). Using Eq. (10), the average RBCv variation was computed showing the progressive but transient increase of RBCv from 3 to 5 mm/s at the end of the stimulation before returning to baseline level (gray plot, Figure 3D). As for the rCBV measure, no RBCv variation associated with the stimuli was detected within vessels located in the ipsilateral S1FL area (black plot, Figure 3D).

Then, the quantification of RBCv variation was extended to all cortical vessels of the brain identified as activated (see Materials and Methods). Thirty-seven cortical vessels were classified as activated with RBCv increase up to 3 mm/s and all spatially located in the S1FL area (Figure 3E). Using the directional component of each activated vessel allows for the discrimination between AV (21%) and PA (79%) involved in the functional response to the forepaw stimulation. Moreover, RBCv in activated AV were slightly increased (peak at 5% increase) in response to the stimuli with a maximum of 25% RBCv increase; however, RBCv change in PA was more distributed with a peak at 10% increase and ranging up to 60% of baseline level (Figure 3F).

Finally, the rCBV signal extracted from the intensity of the Doppler signal and the absolute RBCv from the Doppler frequency were computed together as a third hemodynamic readout to determine relative cerebral blood flow as rCBF = rCBV × RBCv, which provides a more complete description of hemodynamic in each vessel. When processed in the same cortical vessel, it gives an increase of rCBF of ∼100% of baseline level (Figure 3G). Similarly, no variation was observed in the ipsilateral vessels.

## Discussion

Functional ultrasound imaging uses the Doppler intensity as an estimator of the cerebral blood volume (rCBV) (Macé et al., 2011, 2013; Brunner et al., 2021; Edelman and Macé, 2021) without considering the Doppler spectrum, and thus velocity changes, to be independent of the heterogenous profile of vessel distributions in brain voxels. However, by modeling the ultrasound spectrum of the small vessels as a laminar flow and considering the high-pass filter, we allow the absolute quantification of the mean red blood cell velocity (RBCv) in cortical microvessels. Importantly, the RBCv quantification is performed using the same amount of data that are used to compute the intensity image and does not imply any changes of the acquisition protocol.

While this study focuses on cortex-wide hemodynamic changes, the functional ultrasound imaging modality is suited to monitor evoked hemodynamic changes at the whole-brain scale in awake rodents (Urban et al., 2015; Macé et al., 2018; Brunner et al., 2020; Tang et al., 2020; Sans-Dublanc et al., 2021) and potentially, RBCv quantification can be performed in deep subcortical structures in the same way. However, the identification of veins versus arteries in subcortical regions remains challenging since there is no vascular atlas for most species apart from the larger vessels.

The proposed RBCv quantification has some limitations that must be considered during the interpretation of the results. First, we were not able to quantify vessels with average velocity below ∼2 mm/s as they were filtered by the high-pass filter applied to remove tissue motion. Second, as the vessel orientation and the stability of the spectrum are key to adjust the model, one can only apply this strategy in vessels well characterized with prior angiographic image. Third, if the vessel is too short, too small, or simply not linear, it is not possible to measure the angle correctly. Moreover, as the spatial resolution of the angiographic image is ∼100 μm (Macé et al., 2011, 2013; Brunner et al., 2021), only the large size vessels can be detected. With this feasibility study, we identified the vessels manually by using the local maximums in the angiographic image, which is cumbersome and operator dependent. However, this process could be refined and automatized to simplify the procedure.

In our experiment, we identified ∼650 individual cortical vessels/cm^3^ that is approximately a fourth of what can be resolved by the two-photon microscopy (Blinder et al., 2013). This reduction of the detected vessels could be explained by the limitations described previously: a limited spatial resolution and a restriction to address vessels with a RBCv below ∼2 mm/s. Despite these limitations, this tool provides a brain activity with a large-scale coverage not achievable with high-resolution methods such as two-photon microscopy.

Under anesthesia and resting-state conditions, RBCv were ranging from 2 to 12 mm/s for AV and up to 18 mm/s for PA; velocity values are consistent with measures from optical microscopy (Schaffer et al., 2006; Nishimura et al., 2007; Shih et al., 2009, 2012, 2013; Kazmi et al., 2013; Baran et al., 2015; Wang et al., 2019; Ahn et al., 2020; Grubb et al., 2020) and ultrasound imaging (Urban et al., 2014; Errico et al., 2015; Tang et al., 2020; Lowerison et al., 2022). Moreover, cortical vessels located in the contralateral S1FL cortex imaged during electrical forepaw stimulation evoked absolute RBCv changes from 0.5 to 3.0 mm/s with a relative speed increase ranging from 5 to 30% (and up to 60% of baseline level for some individual vessels); changes that are in the line of other studies (Chaigneau et al., 2003; Tiret et al., 2009; Driscoll et al., 2013; Chen et al., 2021). Finally, rCBF, a key parameter of brain physiology, was computed from rCBV and RBCv measurements and depicts more similar functional dynamics than those observed in the previous studies (Liu et al., 2004; Shen et al., 2008; Shih et al., 2011). It is important to note that in this study, the hemodynamics can be affected by the anesthesia (Masamoto et al., 2009; Masamoto and Kanno, 2012), but it can be avoided using awake animals (Urban et al., 2015; Macé et al., 2018; Brunner et al., 2020, 2021; Tang et al., 2020).

It should be noted that focused ultrasound (FUS) has been successfully applied to modulate neuronal activity (see Kamimura et al., 2020 for a minireview on neuromodulation). Nevertheless, functional ultrasound imaging operates in a very different regime of acoustic parameters (higher frequency, shorter pulses, unfocused beams, and low intensity) that are unlikely to cause such effects. In short, these parameters are similar to standard Doppler imaging currently used in the clinics. Moreover, we demonstrated in a recent study with collaborators that neither LFP nor spikes were affected during functional ultrasound imaging (Nunez-Elizalde et al., 2022).

Finally, the accurate measurements of rCBV and absolute RBCv now offered by functional ultrasound imaging modality are of interest to better understand the physiological basis of the neurovascular coupling at the cortex and potential brain-wide scale (Iadecola, 2017; Urban et al., 2017; Bernier et al., 2021) and in pathologies with cerebrovascular dysregulation (Wierenga et al., 2014; Hays et al., 2016), vasculopathies (Itoh and Suzuki, 2012), Alzheimer’s disease (Lowerison et al., 2022), and stroke (Brunner et al., 2017, 2018; Hingot et al., 2020).

## Data Availability Statement

The raw data supporting the conclusions of this article will be made available by the authors, without undue reservation.

## Ethics Statement

The animal study was reviewed and approved by Committee on Animal Care of the Catholic University of Leuven.

## Author Contributions

CB: conceptualization, methodology, formal analysis, investigation, writing—original draft, writing—review and editing, and visualization. EM: conceptualization, methodology, writing—original draft, and writing—review and editing. GM: conceptualization, methodology, software, formal analysis, investigation, writing—original draft, writing—review and editing, and visualization. AU: conceptualization, software, resources, writing—original draft, writing—review and editing, supervision, and funding. All authors contributed to the article and approved the submitted version.

## Conflict of Interest

AU is the founder and shareholder of AUTC company commercializing functional ultrasound imaging solutions for preclinical and clinical research. The remaining authors declare that the research was conducted in the absence of any commercial or financial relationships that could be construed as a potential conflict of interest.

## Publisher’s Note

All claims expressed in this article are solely those of the authors and do not necessarily represent those of their affiliated organizations, or those of the publisher, the editors and the reviewers. Any product that may be evaluated in this article, or claim that may be made by its manufacturer, is not guaranteed or endorsed by the publisher.
