# Supplementary material for: Quantitative Hemodynamic Measurements in Cortical Vessels Using Functional Ultrasound Imaging
Source: Front Neurosci. 2022 Apr 12;16:831650. doi: 10.3389/fnins.2022.831650 (PMC9039668; doi:10.3389/fnins.2022.831650)
Supplement: Supplementary file 1 [file Data_Sheet_1.docx]

**Quantitative hemodynamic measurements at a single cortical vessel resolution in the rat brain using functional ultrasound imaging**

**Supplementary Material.**

**Theoretical spectrum of a small vessel.**

Both particle velocity and Doppler frequency are linked by

C1

where *c* is the speed of sound and the angle between the ultrasound propagation and the velocity.

The velocity within vessels presenting a laminar Poiseuille flow is described as

C2

where *R* is the radius and the maximal velocity at the vessel center.

Considering the annular surface between and , all the red blood cells passing through this surface are contributing with an intensity *dI* to the same Doppler frequency:

with C3

from C1 and C2

, C4

By replacing in C3, we obtain:

with C5

Equation C5 shows that the spectral density is a constant independent of .

**Effect of the transducer bandwidth.**

If the transducer has a emission response , we need to integrate all the contributions of the different ultrasound frequencies, to obtain a simple equation we considerate that the transducer bandwidth is small compared to the ultrasound frequency, the spectrum produced by a single frequency is then considered as a constant independent of

For a given frequency only the ultrasound frequencies higher than contribute to generate this frequency, and thus giving the total spectrum as:

Knowing and the angle of the vessel , one can compute the spectrums for different values of and select the parameter that fits the experimental data.

**Note about the spectral broadening.**

When a particle crosses the point spread function (PSF) there are two main effects modifying the spectrum in a Doppler acquisition sequence.

1) Doppler shift. When the particle crosses the PSF in the z direction (depth) with an angle of 90° the signal is sinusoidal modulated, and the spectrum is shifted (see **Supplementary Figure 1 A, B**).

2) Spectral Broadening. When the particle crosses the PSF in the x direction (horizontal) with an angle of 0° the signal is modulated in amplitude. Its spectrum is enlarged but not (see **Supplementary Figure 1 A, B**).

Effects in the mean frequency.

As the spectral broadening does not shift the frequency the mean frequency is not modified. Only the Doppler effect shifts the frequency. However, we need to use the full spectrum (positive and negative parts) to be sure that the broadening effect is correctly cancellated.

Using a Directional filter.

A directional filter selects only the positive or negative parts of the spectrum. If the spectral broadening is very important, we can bias the result be eliminating some part of the spectrum.

For this reason, we need a minimal angle between the particle and the ultrasound beam to ensure that the Doppler shift is higher than the spectral broadening, and that all the spectrum is placed in the positive (or negative) frequencies.

To compute this minimal angle, we considerate the size of the PSF in the x direction as $dx=\lambda\frac{f}{D}\cong\lambda$ for a standard probe of aperture f/D=1. In the z direction the period is $dz=\lambda/$2 (See **Supplementary Figure 1A**). The Fourier transform of both signals shows that the spectral broadening is 1/4 the Doppler shift. A particle crossing the PSF with an angle sin(o)=1/4 must have a signal with similar length than the horizontal particle. For this reason, using an angle of minimal arcsin(1/4)=15° guarantees that all the spectrum is only in the positive (or negative) frequencies. (See **Supplementary Figure 1C**).

| 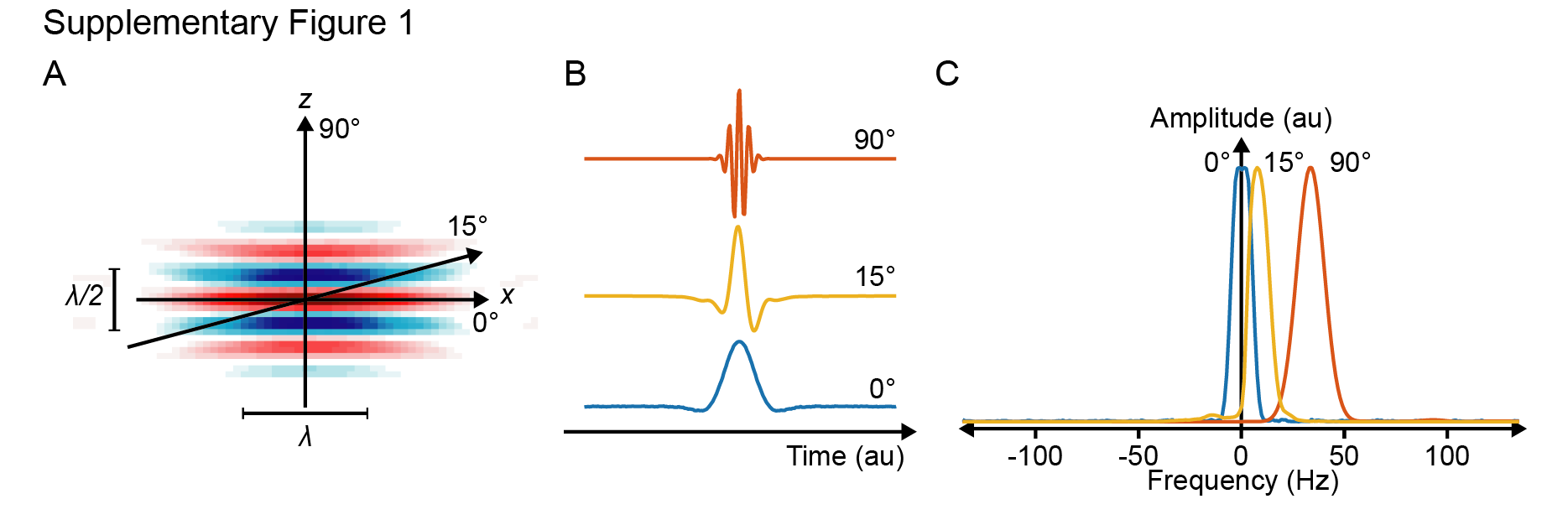 |
| --- |
| **Supplementary Figure 1.** Spectral broadening. **(A)** Point spread function (PSF), **(B)** signal and **(C)** frequencies of particle crossing the PSF with different angle (0, 15, and 90°). |
